# Role of a schistosoma haematobium specific microRNA as a predictive and prognostic tool for bilharzial bladder cancer in Egypt

**DOI:** 10.1038/s41598-020-74807-1

**Published:** 2020-11-02

**Authors:** Dalia A. Gaber, Rita M. Wassef, Wael M. El-Ayat, Mohamed I. El-Moazen, Karim A. Montasser, Sherif A. Swar, Hebat Allah A. Amin

**Affiliations:** 1 Medical Biochemistry & Molecular Biology Department, Faculty of Medicine, Helwan University, Cairo, Egypt; 2 Parasitology Department, Faculty of Medicine, Helwan University, Cairo, Egypt; 3 Medical Biochemistry and Molecular Biology Department, Faculty of Medicine, Ain Shams University, Cairo, Egypt; 4 Urology Department, Faculty of Medicine, Ain Shams University, Cairo, Egypt; 5 Clinical Pathology Department, Faculty of Medicine, Helwan University, Cairo, Egypt; 6 Urology Department, National Institute of Urology and Nephrology, Cairo, Egypt; 7 Pathology Department, Faculty of Medicine, Helwan University, Cairo, Egypt

**Keywords:** Cancer, Molecular biology, Biomarkers, Molecular medicine, Urology

## Abstract

Urinary bladder cancer is a common malignancy in Egypt, thus reliable methodologies are required for screening and early detection. In this study, we analyzed the gene expression of a *Schistosoma hematobium* specific microRNA “*Sha-miR-71a*” and mitogen-associated protein kinase-3 (MAPK-3) in the urine samples of 50 bladder cancer patients and 50 patients with benign bilharzial cystitis. Fifty control subjects were also tested. Indirect hemagglutination test (IHA) diagnosed 70% of studied cancer cases as bilharzial associated bladder cancer (BBC), while histopathological examination detected only 18%. Urinary *Sha-miR-71a* & MAPK-3 revealed enhanced expression in BBC (p-value = 0.001) compared to non-bilharzial bladder cancer (NBBC) cases. Patients with chronic bilharzial cystitis exhibited a significant increase in gene expression compared to those with acute infection (p-value = 0.001). *Sha-miR-71a* and MAPK-3 showed good sensitivity and specificity in the diagnosis of BBC when analyzed by the receiver operating characteristic (ROC) curve. They were also prognostic regarding malignancy grade. Both biomarkers showed a positive correlation. Our results revealed that IHA is a reliable test in the diagnosis of bilharziasis associated with bladder cancer, and that *Sha-miR-71a* and MAPK-3 provide non-invasive specific biomarkers to diagnose BBC, as well as a potential role in testing bilharzial patients for risk to develop cancer.

## Introduction

Bladder cancer is regarded as a common type of malignancy because of its high incidence as well as its elevated relapse rates. In the Egyptian population, this type of cancer is the second most common, particularly among men and it comes as the fourth type among both sexes (7.2%) according to the recent Global Cancer Observatory^1,2^.

Chemical carcinogens are among the most common risk factors in developed countries^3^. Schistosomiasis, which is also known as bilharziasis as well as recurrent urinary tract infection and exposure to radiation are also known to be associated with bladder cancer development in the parasite-endemic areas^4^.

Ancient Egyptians were the first to get acquainted with schistosomiasis. Hematuria was the main complaint of patients with this disease and knowledge about this infestation was mentioned in the Egyptian papyri (1500–1800 B.C.)^5^.

Urinary bilharziasis is caused by *Schistosoma hematobium* trematodes. The manifestations of the disease occur due to the associated immunological reaction produced by the host in response to the worm entrapped eggs. The associated granulomatous tissue reaction affects the urogenital system significantly predisposing bladder cancer^6^. However, we can prevent these chronic sequelae if more knowledge is unraveled about the genetic changes induced by bilharzial infection^7^.

Cystoscopy, although expensive and invasive, is regarded as the gold standard method for diagnosing bladder carcinoma^8,9^. Accordingly, new highly sensitive and specific diagnostic tools, especially those that are urine-based, are particularly striking as urine is a promising and readily available source for molecular markers, such as RNA^9,10^.

MicroRNAs (miRNAs) are key regulators in post-transcriptional suppression. They are small non-coding RNAs, approximately 22 nucleotides in length^11^. Their aberrant expression has been recently used as novel biomarkers detected in the urine of BBC and NBBC patients^12^. Moreover, the role of some small RNA in carcinogenesis and refining prognosis in cell lines have been elucidated^13^. It has been reported that some miRNAs can predict treatment merit and prognosis^10^.

As known as the *Schistosoma* specific miRNAs has an important role in the underlying pathogenesis of schistosomiasis as well as the parasite development, this drew the attention to the possibility of their applications in diagnostics^14^.

Recent studies have provided some valuable information about the small RNA complement of *Schistosoma hematobium*^15,16^. *Sha-miR-71a* is a *Schistosoma hematobium* specific micro RNA with the highest level of transcription in adult worm with no sex bias^16^. Such novel discoveries will facilitate further studies on the parasite genome and host-parasite cross-talk. Early detection for bilharziasis and subsequently decreasing cancer risk is the most efficient strategy for fighting bladder cancer in endemic areas.

Our study is based on the fact that bladder cancer is prevalent in Egypt, with bilharziasis being a neglected disease, though schistosomiasis is an important cause of oncogenesis. Bladder cancer is a preventable cancer if we can reach a non-invasive protocol for early detection, better follow up, and a reliable prognostic marker that can improve management plan and hence an improved outcome. Inspite of the very limited data about the small non coding RNAs sequences of this neglected parasite, we aim that this study and other subsequent studies would enlighten this field of research.

## Patients and methods

### Study design

A multi-center, prospective, cross-sectional study was conducted between February 2019 and January 2020 at the National Institute of Urology and Nephrology, Ain Shams University Hospitals, and Badr Teaching Hospital, Egypt.

### Patient selection

One hundred patients with urinary symptoms (hematuria, urinary retention, dysuria…etc.), who were planned to undergo cystoscopy for suspicion of bladder cancer (BC) or benign bilharzial cystitis (Bil), were selected.

Demographic data and medical history were recorded for each patient. Informed consent was obtained from all participants.

Two minors were included in this study (13&15 year old), and an informed consent was obtained from their parents.

### Study groups

Patients were classified as:

- 50 cases with bladder cancer were chosen based on cystoscopy and histopathologic examination.
- 50 patients with bilharzial cystitis were also included based on positive ova in urine and/or positive IHA tests in collected urine and blood samples respectively.

#### Exclusion criteria

*In bladder cancer group* Patients who have received chemotherapy, or with previously diagnosed other malignancy within the past 5 years.

Control group of 50 healthy volunteers were asked to provide urine and blood samples.

### Samples collection

i. *Urine samples* all subjects were asked to provide urine samples before instrumentation or bladder tumor excision. Freshly voided urine samples (100 ml) were collected in sterile containers.
ii. *Blood samples* 3 ml were withdrawn from patients using sterile labeled vacutainers.
iii. *Tissue samples* forty-eight transurethral cystoscopic resection of bladder tumor (TURBT) samples and two radical cystectomy samples were included.

#### Urine examination

Urine samples were microscopically examined for detection of bilharzial ova.

### Anti-bilharzial antibodies

Blood samples were centrifuged at 3500 rpm for 10 min and sera were kept at – 20 °C for measuring anti-bilharzial antibodies by indirect hemagglutination test (IHA). Cellognost Schistosomiasis H kit, supplied by Dade Behring Marburg GmbH, Marburg, Germany was used^17^.

### Histopathology

Biopsy samples were immediately preserved in 10% neutral buffered formalin and processed for H&E staining for histopathology evaluation. Clinicopathological characteristics of the lesions were reported. These include:

- Type of lesion (inflammatory or neoplastic).
- Presence or absence of bilharzial ova.
- Type of the tumor.
- Stage of the tumor (depth of invasion; if any).
- Grade of the tumor.
- Lymph node status; if any.

### Urinary *Sha-miR-71a* gene expression analysis

#### Sample preparation

The urine samples were centrifuged at 4000 rpm for 20 min in a 15 ml screw-cap tube, containing at least 5 ml of urine, and then the cell pellet was washed with 200 µl of phosphate buffer saline (PBS). The samples were then applied to RNA extraction.

#### PCR primers

*Sha-miR-71a* miScript primer assay was obtained as a custom order; **cat no: MSC0076524**, Qiagen, Germany.

*Hs-miR-16* miScript primer assay was purchased from Qiagen, Germany; **cat no: MSC00031493.**

#### Total RNA extraction and purification

Total RNA was extracted using the miRNA easy Mini Kit; cat no: 217184 (Qiagen, Hilden, Germany) according to the manufacturer’s protocol.

#### Reverse transcription

cDNA was synthesized by reverse transcription reaction using miScript II RT Kit; cat no: 218161; (Qiagen, Hilden, Germany).

#### Sha-miR_71a gene expression analysis

The quantification of miR-71a levels was amplified from miRNA using a miScript primer assays; [miR_71a Primer Assay; ID: MSC0076524]; the miR-16 primer assay, ID: MSC00031493 was used as a housekeeper gene. MiR-71a gene was amplified using miScript Syber green; cat no: 218073 (Qiagen; Hilden, Germany).

### MAPK-3 gene expression analysis

The quantification of the MAPK3 gene was measured using the Hs_MAPK3 _1_SG QuantiTect Primer Assay, cat no: 249900 assay ID: QT02589314 and the QuantiTect SYBR reen PCR Kit cat no: 204141 (Qiagen, Germany). The ACTB Primer sequence; assay ID: QT00095431 as used as housekeeper gene. All samples were analyzed using the 5 plex Rotor-Gene PCR Analyzer (Qiagen, Germany). ACTB as an endogenous reference control for normalization purposes. The 2^−∆∆Ct^ method was conducted for the analysis and measurement of relative gene expression levels^18^.

### Research ethics statement

This study was approved and supervised by the Research Ethics Committee of Faculty of Medicine, Helwan University, Egypt [Serial No: 19/2019].

All research methods were performed in accordance with the relevant guidelines and regulations. An informed consent was obtained from all participants.

### Statistical analysis

Data analysis was performed using Statistical Package for the Social Sciences software (SPSS, Version 19, and Chicago, IL, USA). Quantitative data were summarized using mean, median, standard deviation (SD), minimum and maximum, while frequency/count and relative frequency/percentage were used for categorical data. Comparisons were performed using chi-square, t-tests or ANOVA tests to examine the relation between qualitative variables. *p*-value < 0.05 was considered significant.

## Results

### Patients’data

Sixty-seven men and 33 women were included in this study. The mean age for the cancer group is 61 ± 10 years, while for the bilharzial cystitis group it is 32.5 ± 13. Out of the 50 bladder cancer patients, 80% were males. The clinicopathologic and demographic characteristics are shown in Table 1.

**Table 1:** Demographic and clinicopathologic characteristics of the studied groups.

Variable	Bladder cancer (n = 50)	Bilharzial cystitis (n = 50)	Statistics
**Age (years)**			
Mean ± SD (range)	61 ± 10 (44–75)	32.5 ± 13 (13–55)	F: 0.43; p = 0.55
**Age subgroups (n %)**			
≤ 50	7 (14%)	41 (82%)	χ^2^: 35; p = 0.001*
> 50	43 (86%)	9 (18%)	
**Gender (n %)**			
Male	40 (80)	27 (54%)	χ^2^: 6.3; p = 0.01
Female	10 (20%)	23 (46%)	
**Residence (n %)**			
Rural	0	50 (100%)	χ^2^: 75; p = 0.001*
Urban	50 (100%)	0	
**Presence of ova in urine sample**			
Negative	28 (56%)	21 (42%)	χ^2^: 22; p = 0.001*
Positive	22 (44%)	29 (58%)	

*χ*^*2*^ Chi-square test value, *n (%)* number (percentage).

*Statistically significant difference.

### Microscopic urine examination and serodiagnosis of bilharziasis by IHA

Urine and serological examinations of the samples confirmed the absence of bilharzial infestation in the healthy control group. On the other hand, 70% of patients (35 out of 50), diagnosed with bladder cancer (by cystoscopy and histopathological examination) were positive for anti-Schistosoma antibodies (BBC) by the IHA test, with 51.4% having high antibody titer (> 1/640). Bilharzial ova were detected microscopically in 44% of the bladder cancer group, 86% of which showed positive indirect hemagglutinin test (38% of bladder cancer cases) (Supplementary Fig. S1).

Regarding the bilharzial cystitis group, bilharzial ova was detected in 58% of cases and IHA was found positive in 42% (Table 1).

### Histopathological examination

#### Cystitis group

**Figure 1:**
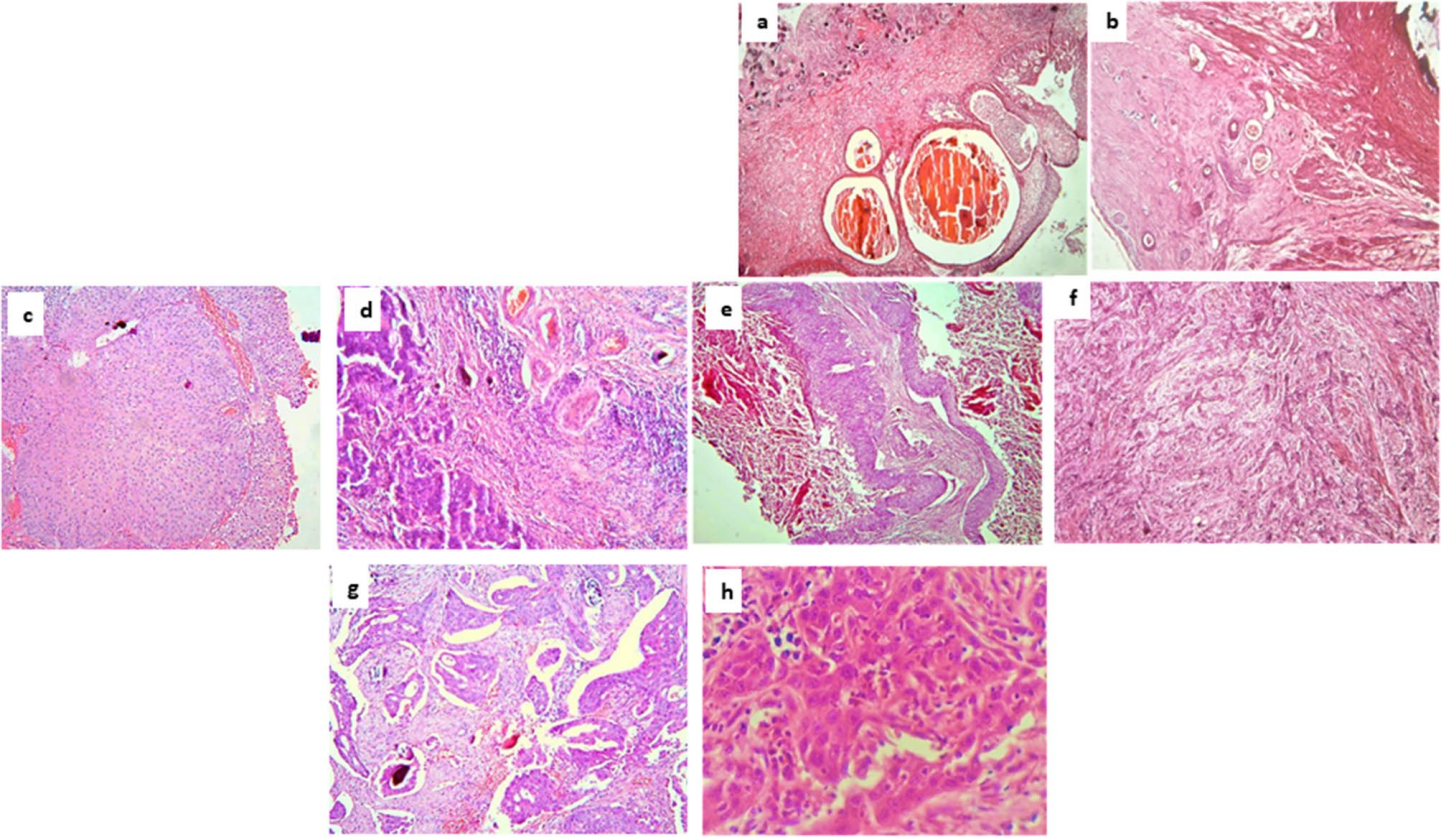
Histopathological examination. (**a**) Bilharzial proliferative cystitis with cystitis cystica and cystitis glandularis associated with moderate dysplasia (TUBT). (**b**) Bilharzial seminal vesiculitis in cystectomy specimen for TCC. (**c**) Low grade TCC with bilharzial ova (TURBT). (**d**) High grade TCC with bilharzial ova (cystectomy). (**e**) High grade TCC with bilharzial ova (cystectomy). (**f**) Low grade TCC with encrustations and no bilharzial ova (TURBT). (**g**) SCC with histopathologic evidence of Schistosomiasis (cystectomy). (**h**) SCC with no reported ova.

In inflammatory mass lesions, Schistosoma ova were histologically detected in only nine cases. While features of proliferative cystitis with Brunn’s nests, cystitis cystica, and cystitis glandularis, with, or without, dysplasia were noted in 50 cases (Fig. 1).

#### Bladder cancer group

Forty-eight samples were provided by transurethral resection of bladder tumor (TURBT) while two were radical cystectomy specimens. Six cases were pure squamous cell carcinoma (SCC), four low-grade superficial, non-invasive papillary transitional cell carcinoma and 40 cases high-grade transitional cell carcinoma (TCC), High grade TCC attributed to 80% of the cancer cases, six of which were associated with squamous features (Fig. 1).

Regarding the staging, seven cases were Ta, only one case was T1, 34 cases were T2, and 8 cases were T3b. As to the nodal status, the two radical cystectomy cases were representing 80% of the cancer cases, six of which show associated squamous features (Fig. 1).

Regarding the staging, seven cases were Ta, only one case was T1, 34 cases were T2, and 8 cases were T3b. As regards nodal status, the two radical cystectomy cases were N0. Bilharziasis was histologically confirmed in only 9 cases (18%).

### Urinary *Sha-miR-71a* gene expression levels in the studied groups

**Figure 2:**
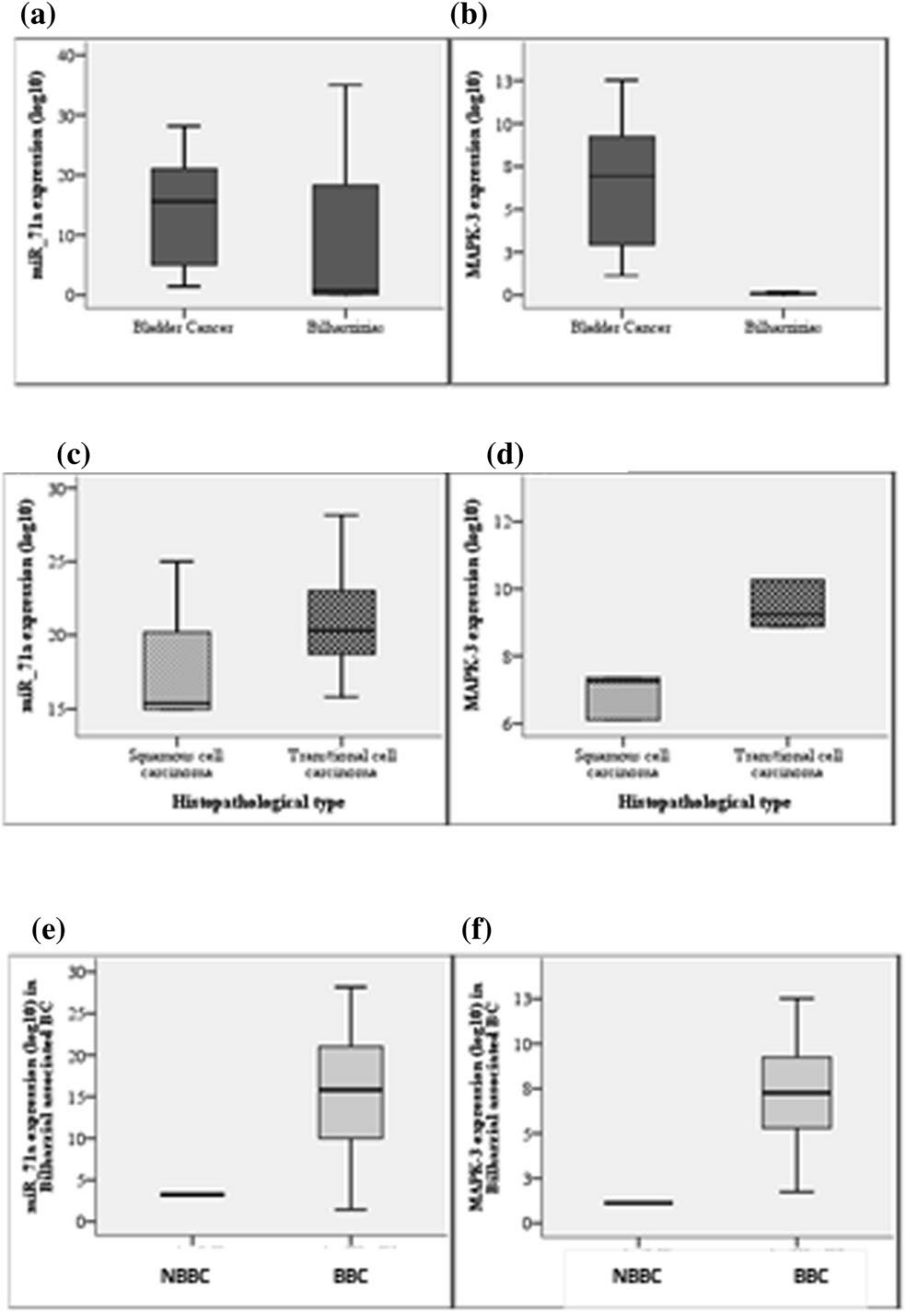
Comparison of gene expression of sha-miR-71a & MAPK3 among studied groups. (**a**) Show a significant increase in gene expression of Sha-miR-71a in BC group compared to bil. cystitis group, U = 436, P = 0.003[HS]. (**b**) Show a significant increase in gene expression of MAPK3 in bladder cancer group compared to bil. cystitis group, U = 0.00, p = 0.001[HS]. (**c**) show an enhanced gene expression of Sha-miR-71a in patients with transitional cell carcinoma compared to those with squamous cell carcinoma, U = 186, p = 0.04[S]. (**d**) Show an increased gene expression of MAPK3 in patients with transitional cell carcinoma but not significantly different from squamous cell carcinoma, U = 202, p = 0.08[NS]. (**e**) Show a significant increase in gene expression of Sha-miR-71a in BBC group compared to NBBC, U = 0.00, p = 0.001[HS]. (**f**) Show a significant increase in gene expression of MAPK3 in BBC group compared to NBBC, U = 0.00, p = 0.001[HS]. *U* Mann Whitney test, *HS* highly significant difference, *S* significant difference, *NS* non significant difference, *BC* bladder cancer, *BBC* bilharzial bladder cancer, *NBBC* non bilharzial bladder cancer, *Bil.* bilharzial cystitis.

Comparing the expression levels of miR-71a (indicated by RQ values) between studied groups, the BC group showed higher RQ level (median: 15.8), compared to benign bilharzial cystitis group (median: 0.73) with significance value p = 0.003. Comparing gene expression levels of miR-71a in BBC and NBBC, a highly significant difference was noted, with enhanced expression in the BBC group. Within the bladder cancer group, transitional cell carcinoma cases showed enhanced gene expression of *Sha-miR-71a* compared to those with squamous cell carcinoma, and cases with high-grade malignancy varied significantly in gene expression of *Sha-miR-71a* compared to those with low-grade malignancy (Fig. 2).

Urinary *Sha- miR-71a* was more expressed in patients with chronic bilharzial infection, and in those with high IHA titer compared to those with recent infection and low antibody titer respectively, showing a highly significant difference (p = 0.001) (Table 2).

**Table 2:** Comparative analysis between the expression level of miR-71a and MAPK-3 in patients with bilharzial cystitis.

Groups	Sha-miR-71a (log^10^)		MAPK-3 (log^10^)	
	Median (range)	Statistical analysis	Median (range)	Statistical analysis
**Bilharzial cystitis**				
Acute infection (IHA negative) (n = 29)	0.1 (0.07–15.3)	χ^2^ = 15.3 p = 0.001[HS]	0.05 (0.03–0.1)	χ^2^ = 16.2 p = 0.001[HS]
Chronic infection (IHA positive) (n = 21)	21.3 (0.07–35)		0.16 (0.03–0.2)	
**Bilharzial antibody titer**				
≤ 1/640 (n = 9)	15.4 (15.4–22)	χ^2^ = 13.6 p = 0.001[HS]	0.1 (0.1–0.2)	χ^2^ = 6.9 p = 0.008[HS]
> 1/640 (n = 12)	35 (21.2–35)		0.2(0.16–0.2)	

*n* number, *χ*^*2*^ Chi-square test value, *HS* highly significant difference, *S* significant difference, *NS* non significant difference.

### Urinary MAPK3 gene expression levels in the studied groups

MAPK-3 gene was significantly expressed in the bladder cancer group, compared to bilharzial cystitis group. Testing the differential gene expression of MAPK-3 in BBC and NBBC, a highly significant difference was noticed with enhanced expression in the BBC group. Within the bladder cancer group, transitional cell carcinoma cases had enhanced gene expression of MAPK-3 compared to those with squamous cell carcinoma, and cases with high-grade malignancy varied significantly in gene expression of MAPK-3 compared to those with low-grade malignancy (Fig. 2).

Urinary MAPK-3 was more expressed in patients with chronic bilharzial infection, and in those with high IHA titer compared to those with recent infection and low antibody titer respectively, showing a highly significant difference (p value = 0.001) (Table 2).

**Figure 3:**
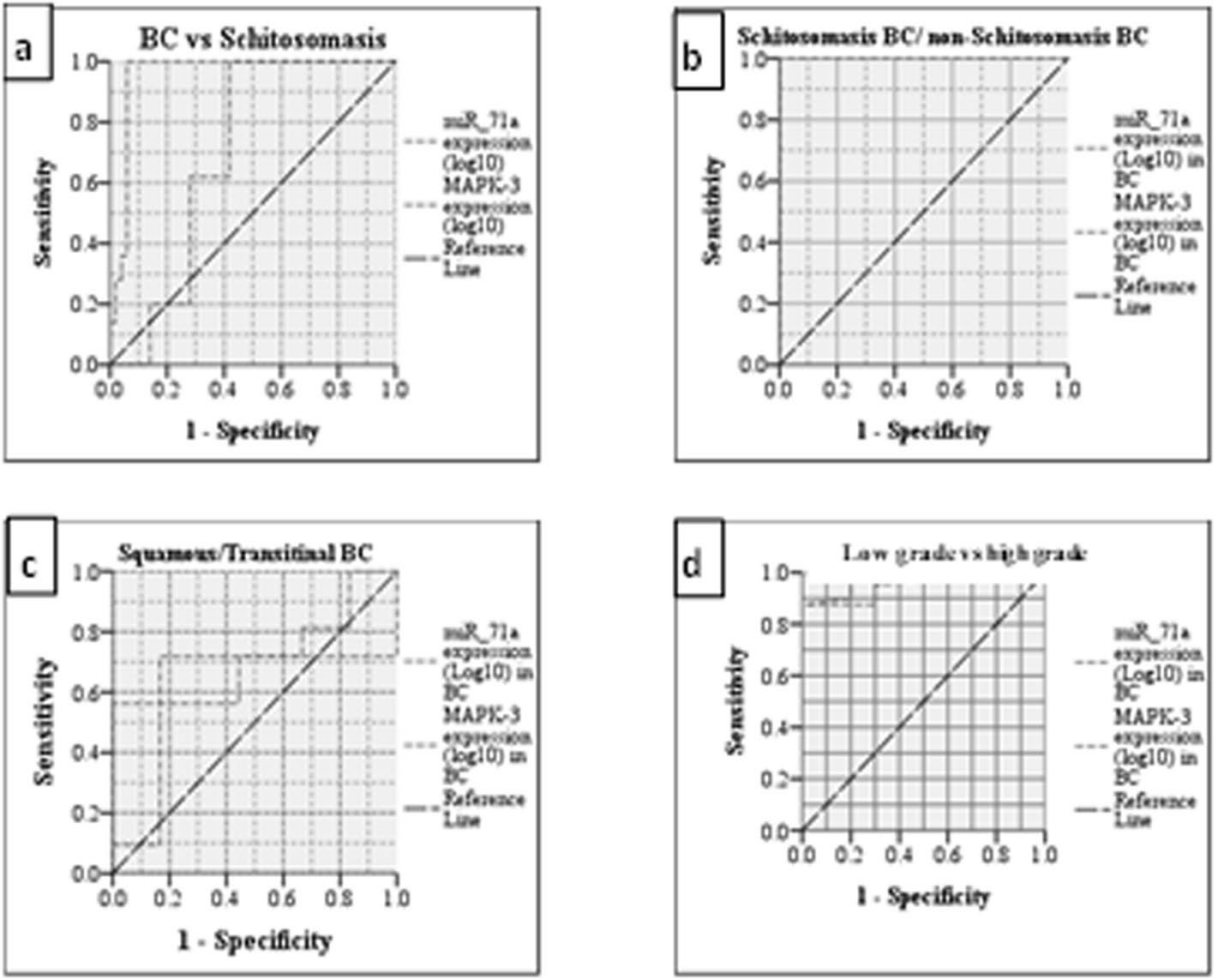
ROC curve analysis for Sha-miR-71a & MAPK-3 genes. (**a**) Discriminating malignant from non-malignant group, best cut off value for miR-71a = 15.0 [sensitivity = 70% and specificity = 72%], area under the curve (AUC) = 0.6, 95% confidence limits range = 0.581–0.809, P 0.01, for MAPK-3 = 2.1 [sensitivity = 88% and specificity = 94%]. AUC = 0.95, 95% confidence limits range = 0.9–1.0, P = 0.001. (**b**) Discriminating BBC from NBBC, best cut off value for miR-71a = 10.5 [sensitivity = 88% and specificity = 100%], area under the curve (AUC) = 1.0, 95% confidence limits range = 1.0–1.0, P = 0.001, for MAPK-3 = 6.3 [sensitivity = 87% and specificity = 100%]. AUC = 1.0, 95% confidence limits range = 1.0–1.0, P = 0.001. (**c**) To differentiate squamous from transitional cell carcinoma, for miR-71a = 10.2 [sensitivity = 72% and specificity = 84%], area under the curve (AUC) = 0.67, 95% confidence limits range = 0.51–0.84, P 0.039, for MAPK-3 = 0.65 [sensitivity = 72% and specificity = 56%], AUC = 0.65, 95% confidence limits range = 0.49–0.805, P = 0.082. (**d**) To differentiate low-grade from high-grade bladder cancer. Best cutoff point for miR-71a = 10.0 [sensitivity = 87% and specificity = 99%], area under the curve (AUC) = 0.97, 95% confidence limits range = 0.93–1.0, P 0.001. for MAPK-3 = 5.2 [sensitivity = 88% and specificity = 99%], AUC = 0.93, 95% confidence limits range = 0.89–1.0, P = 0.001.

Analysis of *Sha-miR-71a* expression by the receiver operating characteristic curve (ROC), *Sha-miR-71a* had a sensitivity of 70, 88, 72, and 87% and a specificity of 72, 100, 84, and 99% for diagnosis of BC, BBC, transitional carcinoma, and high grade malignancy respectively. The areas under the curve (AUC) were 0.695, 1.0, 0.677, and 0.97 and cut-off points were 15.0, 10.5, 10.2, and10 for diagnosis of BC, BBC, transitional carcinoma, and high grade malignancy respectively (Fig. 3).

MAPK-3 analysis showed a sensitivity of 88, 87, 72, and 88% and specificity of 94, 100, 56, and 99% for diagnosis of BC, BBC, transitional carcinoma, and high grade malignancy respectively. AUC was 0.956, 1.0, 0.65 and 0.95 and cut-off points were 2.1, 6.3, 5.2, and 5.2 for diagnosis of BC, BBC, transitional carcinoma, and high grade malignancy respectively (Fig. 3).

**Figure 4:**
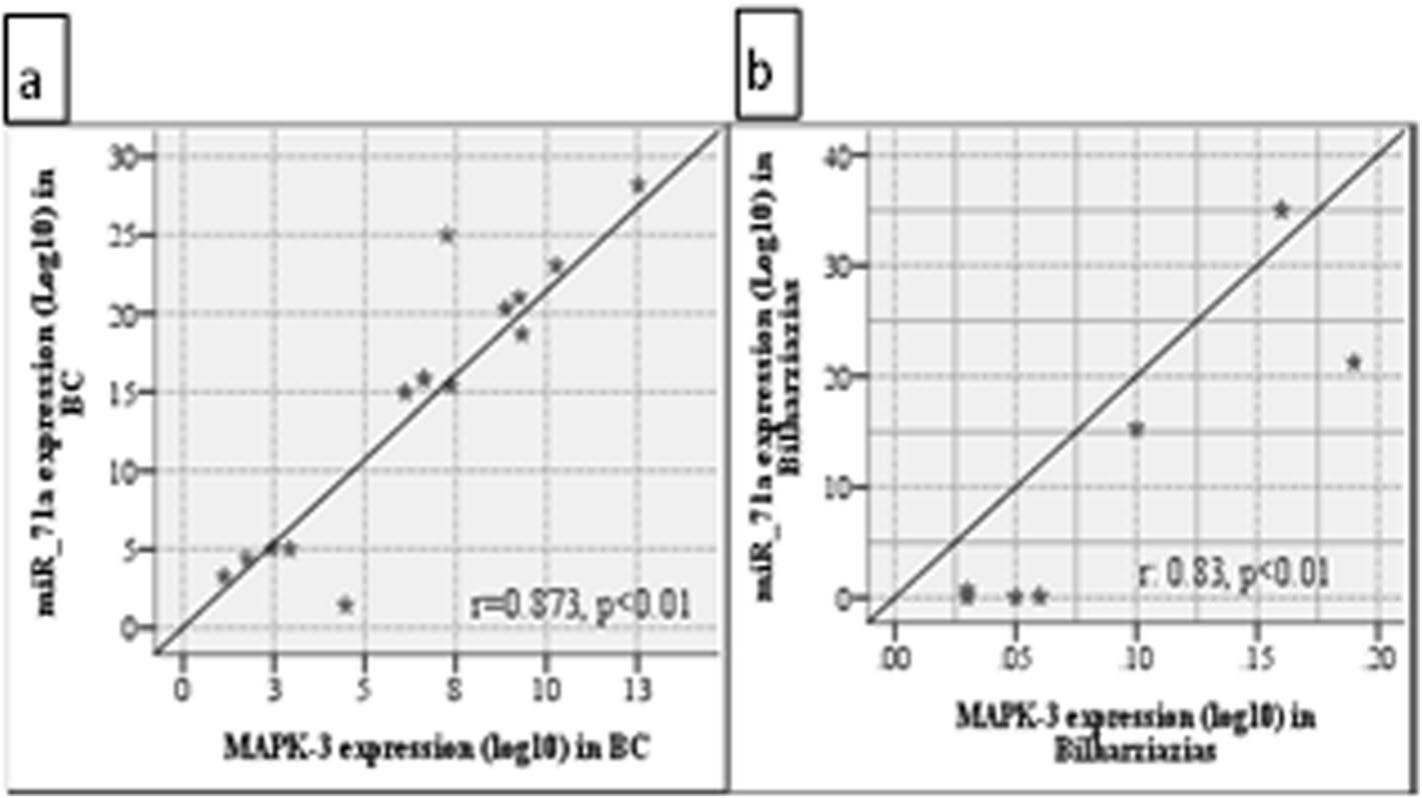
Scattered plot analysis of Pearson’s correlation between sha-miR-71a expression & MAPK-3 expression. (**a**) shows that there is strong correlation between the expression of both genes in the bilharzial bladder cancer group, with r = 0.83, p < 0.01. (**b**) Shows that there is strong correlation between the expression of both genes in the bilharzial cystitis group, r = 0.873, p < 0.01.

Pearson’s correlation analysis showed a strong correlation between *Sha-miR-71a* and its gene target MAPK-3 in both the bilharzial bladder cancer group and the bilharzial cystitis group, (Fig. 4).

## Discussion

Bladder cancer in Egypt has its unique features, in part because of *Schistosoma hematobuim’s* infestation. Its high prevalence requires the continuous search for more specific, accurate and non-invasive biomarkers for early detection and better prognostic outcomes.

There are several misbeliefs concerning its association with bilharziasis, and subsequently the lines of management^19^. Published researches in Egypt are either old studies suggesting the prevalence of SCC^20^, recent studies claiming control of schistosomiasis^21^, or recent studies suggesting that there is no difference between BBC and NBBC cancer regarding disease pathogenesis, prognosis, and management^22–24^.

Hereby, we have decided to search for the possible defects in the methodology of previous studies and to fill the literature gaps that have led to unreal proposed facts affecting our daily practice.

Firstly, urine and blood samples were collected from patients suspected of bilharzial bladder cystitis, attending the urology clinic in Badr Teaching Hospital, National Urology Institute, and Ain Shams Teaching Hospitals. Only those with positive ova in urine and/or positive IHA tests were included in this group, which reached 50 samples. After centrifugation of urine samples, the cell pellet was washed with PBS and kept at − 80 °C after adding RNAlater (Qiagen, USA) for further genetic study.

Microscopic examination of urine samples confirmed the presence of ova in 29 out of the 50 patients. The other 21 cases showed positive IHA tests, with no ova detected by microscopic urine examination. Accordingly, this group was sub-classified into acute and chronic cystitis. We postulate that these 2 tests should be done as a screening, complementing each other in endemic areas to cover the spectrum of acute and chronic infestations. It is noteworthy that 100% of these patients came from rural areas. Only nine cases were histologically confirmed as bilharzial in the cystoscopic biopsies reflecting the marginal role of histopathology in the grouping of cases as bilharzial and non-bilharzial.

In our study, cases of bilharzial cystitis included two 13 and 15-year old boys reflecting new bilharzial infestations. Noteworthy, our cases were referred to hospitals in Cairo, where the possibility of receiving patients of rural residency acquiring this infestation is much less than in other rural hospitals. This should draw attention to the importance of having a national screening program. There should be a sensitive method to detect the actual burden of bilharziasis in Egypt, especially with the persistent prevalence of bladder cancer^19^.

Secondly, we collected urine and blood samples from patients who were candidates for cystoscopic resection/biopsy due to suspicion of bladder cancer (hematuria and bladder mass by U/S). Samples of this group were for patients attending urology clinics at the National urology institute and Ain Shams teaching hospitals. Cystoscopic specimens were histopathologically examined and 50 cases with proved bladder cancer were included in the study. The majority of cancer cases (80%) were males. This is aligned with other studies confirming the prevalence of such neoplasia among males^19,25^. As regards the age, cancer was reported about three decades later than that of the cystitis group (61 ± 10 years for the cancer group versus 32.5 ± 13 for the cystitis group). This is attributed to the long term complication of the chronic inflammatory process. This comes in agreement with previous data about the incidence of bladder cancer which increases with age, particularly between the age of 50 and 70 years^26^.

Also, all our gathered cancer cases presented from urban areas, while patients presenting with the inflammatory lesions lived in rural areas. This could be attributed to the fact that bilharzial infestation requires an agricultural environment. Mass immigration from rural to urban areas can explain the presence of cancer in urban areas (Table 1).

Based on the results of the IHA test, cancer patients were classified as BBC and NBBC^17,19^. It is worthy to note that histopathologic confirmation of bilharziasis is reported in only 18% of cancer cases in comparison with 70% by IHA. This again reflects the poor sensitivity of histopathology as a diagnostic tool for bilharziasis^19^.

Using more than one method for the confirmation or exclusion of bilharziasis in the inflammatory and neoplastic cases brings a higher degree of accuracy in the categorization of the cases as bilharzial and non-bilharzial. This is different from what is published in the recent Egyptian articles. This variable was frequently neglected though it is an important parameter in the methodology^22^.

Limited knowledge of the genome of *S. haematobium* has for long hindered the discovery of this parasite which is considered neglected, compared to other schistosomes. One of the earliest studies in 2011^27^ was able to silence two genes in *S. haematobium* using RNA interference. Such experiment pointed that it is possible to manipulate the parasite genes for the purpose of study and search for novel anti-parasite treatments.

The identification of molecular biomarkers like miRNAs has proved to be of value in recent years since genetic and epigenetic dysregulations are regarded as the main contributors in the initiation and progression of cancer^28^. Biomarkers have been approved by the FDA to be used in the detection of bladder cancer. However, one parameter is not enough to provide clinicians with the necessary information^29^.

Increased knowledge of *Schistosoma hematobium* small non-coding RNA sequences^16^ may provide insights into the parasite-host interactions which might enable the development of novel strategies to lessen its deleterious pathogenesis and progression to malignancy.

In this study, gene expression of a *Schistosoma hematobium* derived micro RNA “*Sha-miR-71a*” and its gene target MAPK-3 mitogen-activated protein kinase-3^16^ was measured in urine samples of patients diagnosed with bladder cancer (BBC and NBBC) as well as patients with bilharzial cystitis using real-time reverse transcription RT-PCR. To our knowledge, this study is the first to investigate a *Schistosoma hematobium*-specific small non-coding RNA and its role in the pathogenesis of chronic cystitis and bladder cancer.

*Sha-miR-71a* is the most highly transcribed small non-coding microRNA in both males and females adult worm^16^. Using bioinformatics tools, miRanda v.3.3a^29^ and PITA^16^, the gene targets of this microRNA in the host were determined and mitogen-activated protein kinase -3 “MAPK-3” was selected. This gene target was chosen because dysregulated MAPK pathway is involved in bladder cancer^30^. Also, Miranda 3.3a score was > 300 and PITA score was < − 10 indicating valid binding sites^16^. To assess its expression level in patients enrolled in this study. MAPK is known to have aberrant gene expression in bladder cancer and to play a role in tumorigenesis^10^. This raises the question about the role of *Sha-miR-71a* in the initiation of cancer by targeting the mitogen-activated protein kinase, a protein that enhances cellular proliferation.

Using the 2^−∆∆Ct^ method, relative gene expression was calculated. Both genes (*Sha-miR-71a* and MAPK-3) were found to be significantly expressed in cancer cases more than benign cystitis cases (Fig. 2). A highly significant difference was noted in the expression level of both genes between BBC &NBBC with enhanced expression in the BBC group. This indicates its specificity in the diagnosis of bilharzial bladder cancer.

Within the bladder cancer group, transitional cell carcinoma cases had enhanced gene expression of *Sha-miR-71a* and MAPK-3 compared to those with squamous cell carcinoma. This reflects the changing pattern of bladder cancer in Egypt because the prevalent cancer in our cases is TCC (88%) where SCC is progressively declining. We do not believe that this is attributed to the control of bilharziasis for several reasons. Firstly; even with efficient control; the previous burden of bilharzia infestation a few decades before will lead to the emergence of new cancer cases nowadays. Secondly, new fresh cases are still being reported. Thirdly, 88% of patients who were classified as BBC were transitional cell carcinoma, and lastly, the expression of schistosoma specific microRNA (*Sha-miR-71 a*) was significantly increased in TCC cases compared to its expression in SCC cases. Also, noteworthy to mention that nodal metastasis was not reported in any of our cases. This could be attributed to the limiting host immune response^31^.

We also noticed that cases with high-grade malignancy varied significantly in gene expression of *Sha-miR-71a* and MAPK-3 compared to those with low-grade malignancy (Fig. 2) and expression levels were increased in high grade cancer (RQ value was significantly different from low grade cancer cases). This draws the attention to this micro RNA as having a prognostic value.

We should note here that the median RQ of both *Sha-miR-71a* and MAPK-3 significantly increased in cases with chronic bilharzial cystitis (cases with positive IHA and no ova in urine) compared to acute cases (Table 2), with MAPK-3 significantly rising in BBC (Fig. 2). This means that MAPK-3 and epigenetic regulator *sha-miR-71a*, both must be elevated to diagnose BBC. This finding can direct us to validate a protocol that ensures close follow up of cases with bilharzial cystitis for prevention and early cancer detection.

The ROC curve analysis revealed urinary *sha-miR-71a* and MAPK-3 to be diagnostic for BBC at a cut off 10.5 and 6.2 with 88%, 87% sensitivity and 100%, 100% specificity, respectively. They were also of prognostic value regarding malignancy grade at a cut off 10.5 and 6.2 with 87%, 88% sensitivity and 99%, 99% specificity, respectively. Both urinary biomarkers were found to be statistically correlated. They can be potential biomarkers providing a non-invasive, easy and specific tool that can be of diagnostic and prognostic value.

In previous studies^7,10,12^, genomic instability due to schistosomiasis and aberrant miRNAs produced by the host in response to schistosomiasis infection were studied. Thus such epigenetic regulators mediate disease pathogenesis and its carcinogenesis and can be used as potential biomarkers^32^. Another study was performed on *Schistosoma japonicum* miRNAs, their role in cancer initiation and how this can lead to new interventional strategies to halt ongoing pathogenesis^33^.

Dysregulated miRNAs and their possible targets involved pathogenesis of muscle-invasive bladder cancer have also been identified, which can be of value in modifying therapeutic strategies^10^. A miR-192-5p was described as a promising therapeutic target since its overexpression inhibited the growth of bladder cancer cells^32^.

In another study, urinary sFas was pointed to as a non-invasive diagnostic marker for BBC^34^. In a study in 2018^13^, a circular RNA (Cdr1) was used for therapeutic purposes, by targeting miR-135a, up-regulating p21 and thus decreasing proliferation of bladder cancer cells.

Also another study^35^ suggested that aberrant DNA methylation of cancer genes, like RARb2 and APC can be of value as urinary molecular markers for early detection of bilharzial and non bilharzial bladder cancer.

## Conclusion and recommendations

1. The oncogenic role of bilharziasis is underestimated in Egypt.
2. There is a changing pattern of bladder cancer in Egypt with increased levels of TCC and decreased levels of SCC. Schistosomiasis remains a major cause of tumorogenesis.
3. BBC is a preventable disease if reliable tools for screening are provided.
4. It is highly recommended to implement a national screening program for schistosomiasis with subsequent screening and continuous follow up of those with increased cancer risk. This approach could decrease the burden of bladder cancer in Egypt.
5. A pilot study on a small scale can provide the required needs as an assessment for a national program.
6. Urinary MAPK-3 &its epigenetic regulator, *Sha-miR-71a* can provide diagnostic non-invasive specific markers to differentiate BBC from NBBC.
7. A suggested plan for screening patients with cystitis is as follows: Perform IHA and microscopic urine examination, followed by urinary *Sha-miR-71a* and MAPK-3 genetic testing for positive cases. High expression levels should be considered at risk to develop cancer.

## Supplementary information

Supplementary information 1.

Supplementary information 2.
