# Supplementary figures and images for: Role of a schistosoma haematobium specific microRNA as a predictive and prognostic tool for bilharzial bladder cancer in Egypt
Source: Sci Rep. 2020 Nov 2;10:18844. doi: 10.1038/s41598-020-74807-1 (PMC7606480; doi:10.1038/s41598-020-74807-1)

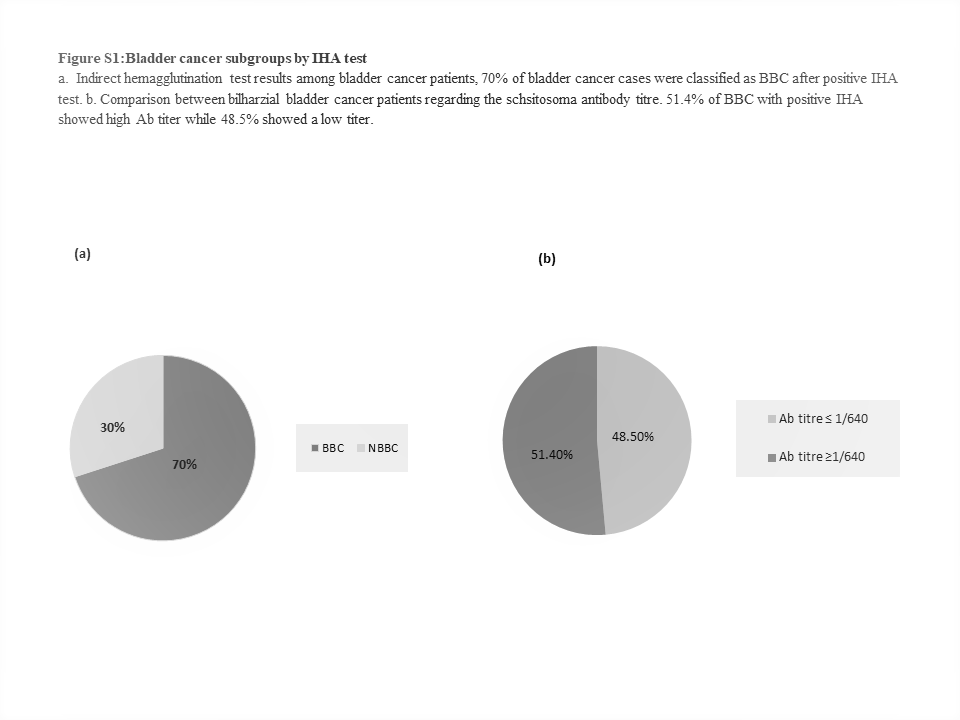

Supplement: Supplementary file 1 — Supplementary information 1. [file 41598_2020_74807_MOESM1_ESM.tif]

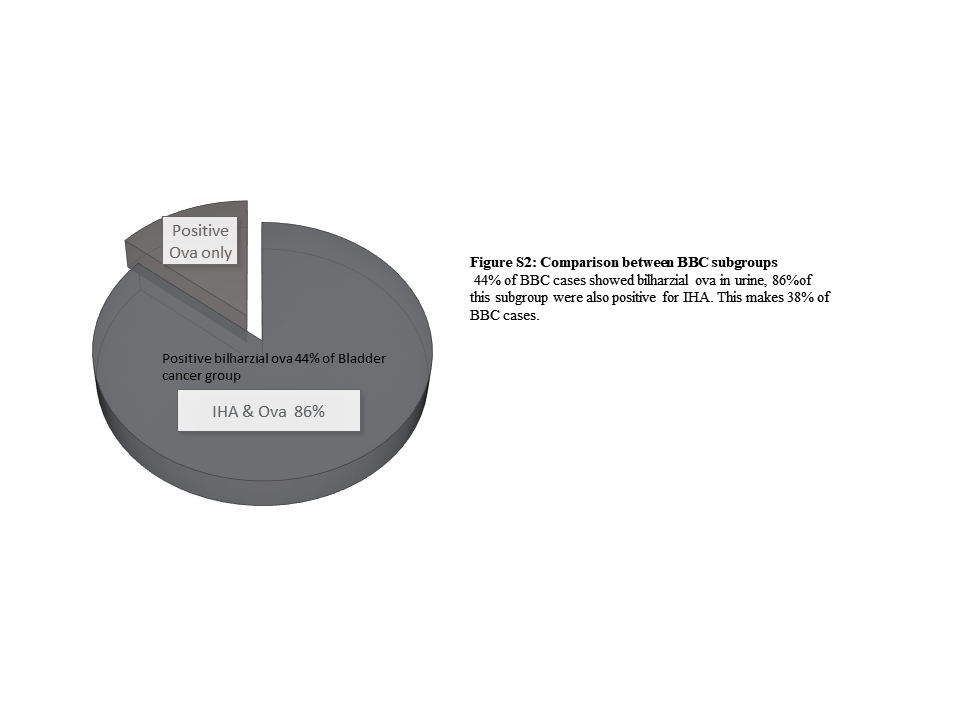

Supplement: Supplementary file 2 — Supplementary information 2. [file 41598_2020_74807_MOESM2_ESM.tif]
